# Evaluation and analysis of anxiety and depression symptoms for college students during COVID-19 pandemic

**DOI:** 10.1186/s40359-022-00934-1

**Published:** 2022-09-30

**Authors:** Dingwei Gao, Qingzhi Xiang, Ganghua Lu, Junyu Tong, Wen Jiang, Xiaqing Yu, Ru Wang, Zhongwei Lv, Dan Li

**Affiliations:** 1 Department of Nuclear Medicine, Shanghai Tenth People’s Hospital, School of Medicine, Tongji University, Shanghai, 200072 China; 2 Department of Spine Surgery, Tongji Hospital, Tongji University School of Medicine, Shanghai, 200065 China; 3 Department of Nuclear Medicine, Sun Yat-sen Memorial Hospital, Sun Yat-sen University, Guangzhou, 510289 China

**Keywords:** College students, COVID-19, The GAD-7 and PHQ-9 scale, Reliability and validity, Anxiety and depression symptoms, Mental health

## Abstract

**Background:**

The mental health of students is affected by COVID-19. We aim to evaluate the anxiety and depression symptoms among college students during COVID-19 pandemic, analyze the influence factors that contribute to college students’ anxiety and depression symptoms, and provide some suggestions for improving the mental health of college students.

**Methods:**

With 179 college students participating, an online questionnaire consisting of a general questionnaire, Generalized Anxiety Disorder (GAD-7), and Patient Health Questionnaire (PHQ-9) was conducted in universities in Shanghai. The anxiety and depression symptoms among college students were evaluated using GAD-7 and PHQ-9 scales, and influence factors were analyzed using an unordered multi-class Logistic regression model.

**Results:**

The reliability and validity of the GAD-7 and PHQ-9 scales were good (reliability ≥ 0.9, validity = 100%). The incidence of anxiety was 32.4%, of which were 23.5%, 8.4%, and 0.6% in mild, moderate, and severe, respectively; and the incidence of depression was 46.40%, of which in mild, moderate, moderate to severe, and severe were 28.5%, 10.1%, 7.3%, and 0.6%, respectively. Multivariate analysis revealed that male students with strong psychological quality, who were not easily affected by the COVID-19 pandemic, who received less negative or false information, and who had a strong grasp of psychology and related knowledge were less likely to suffer from mild or moderate anxiety symptoms [OR (95% CI) 0.18 (0.04, 0.81), 0.12 (0.05, 0.33), 0.23 (0.06, 0.89) and 0.07 (0.01, 0.74)]. Furthermore, college students who were not affected by the COVID-19 pandemic were less likely to suffer from mild, moderate, and moderate to severe depression symptoms [OR (95% CI) 0.23 (0.08, 0.65), 0.22 (0.05, 0.93), 0.10 (0.02, 0.54)].

**Conclusion:**

The GAD-7 and PHQ-9 scales are suitable for evaluating anxiety and depression symptoms in college students. The COVID-19 pandemic was associated with a high incidence of anxiety and depression symptoms among college students, although gender and mental state fluctuations during the pandemic, negative and false information, and exposure to psychology and related courses were the main influencing factors.

**Supplementary Information:**

The online version contains supplementary material available at 10.1186/s40359-022-00934-1.

## Introduction

In December 2019, COVID-19 was first discovered in Wuhan, Hubei Province, and rapidly spread to the rest of China and hundreds of other countries and regions [1–4]. On January 30, 2020, the World Health Organization declared the COVID-19 pandemic an international health emergency [5]. With the domestic attention to the pandemic situation and the implementation of control measures, the domestic pandemic situation has improved, but the international pandemic situation continues to rage [6, 7]. COVID-19 poses enormous risks, as it not only aggravates the condition of patients with underlying diseases and accelerates death in the elderly [8–10], but also has many long-term symptoms that cannot be ignored, such as fatigue, muscle, and joint pain, lung abnormalities, abnormal liver, and kidney function, loss of smell and taste, anxiety and depression [11, 12].

The COVID-19 pandemic causes serious health problems for COVID-19 patients, medical staff, and their quarantined observers, and the general public is also prone to severe panic, anxiety, and unease [13–15]. On the one hand, COVID-19 infection is sudden, has a very strong pandemic, widespread transmission, lack of specific drugs, and high mortality in high-risk groups; COVID-19’s characteristics have caused public panic, anxiety, depression, and unease [14]. On the other hand, excessively negative information about the pandemic or the lack of psychological relief measures for public emergencies also aggravates the public’s negative psychological emotions or symptoms [16]. Moreover, different countries hold different coping strategies in the face of such an unexpected public event. Most Western countries, mainly the United States, have an open attitude and a herd immunity policy [17]. On the contrary, China has been particularly active in its response and has achieved relatively positive results [18, 19]. However, due to the aggressive travel restrictions, the flow of people and goods has been reduced, substantially impacting economic and social development [20–22]. In short, the pandemic has direct or indirect effects on the physical and mental health of the public.

Public health events occurring during this period are highly likely to trigger higher levels of generalized anxiety and depression among college students during their active stage before entering the campus [23–25]. To stop the spread of the COVID-19 pandemic to the campus, the Ministry of Education requires that the start of the spring semester in 2020 be postponed. For college students, the holidays are extended, they stay at home for a long time, they must go out less, and they are unable to attend school normally to study and participate in social activities, which may affect their studies and aggravate their anxiety and depression. Many studies have also focused on the mental health of large study groups during the epidemic. Among the factors studied, there is a lack of sufficient research focusing on the popularity of psychology-related courses, control policies, and negative information on anxiety and depression of university student groups during the epidemic [23, 25].

The present study is a cross-sectional study to understand the general objective psychological performance of college students in the current epidemic environment and under China’s unique active blockade policy and the association of specific control policies and psychological knowledge with anxiety and depression symptoms. To rationalize society’s concern about the mental health status of college students when the pandemic was expected to improve but potentially worsen. As well as the subsequent resurgence of the pandemic, it is possible to accurately, purposefully, and effectively find and develop interventions to reduce anxiety and depressive symptoms in the college student population and effectively reduce the risk and trend of these negative emotions getting worse.

## Methods

### Study design

The primary objective of the present study approach was to confirm there was a high incidence of anxiety and depression symptoms among university students with COVID-19 Pandemic, and to find some factors that affect the anxiety and depression symptoms of college students.

The sample size was calculated using the normal approximation of a single group rate. By calculation (Additional file 1), we would require 188 cases of participants, and in reality, we collected 185 data, which was approximate to our expected sample size. We finally screened the sample to 179 cases due to missing data or unqualified filling quality.

### Participants

After obtaining the approval of relevant institutions, an online questionnaire was randomly distributed to all grades of universities in Shanghai using “questionnaire Star” (address: https://www.wjx.cn/) from March 9 to March 12, 2020. The occupation was limited to college students.

### Inclusion and exclusion criteria

Inclusion criteria: students in universities in Shanghai, no restrictions on majors, no restrictions on regions, and voluntary survey is preferred.

Exclusion criteria: if the filling time of the questionnaire is less than 3 min and the completion of the questionnaire is less than 100%, those who fill in the questionnaire during the non-survey period or those surveyed are non-college students, and those who are unwilling to accept the survey will be excluded.

### Method and content

The online questionnaire included a general situation questionnaire, a generalized anxiety disorder scale (GAD-7) [26], and a Patient Health Questionnaire scale (PHQ-9) [27–29].

We designed the general questionnaire, and the main contents include age, gender, current health status, familiarity with psychology and related knowledge, attention to COVID-19, and the impact of COVID-19. The recent health status item was designed to determine whether the invitee was suffering from physical illness, with three scales: healthy, isolated (good health), and poor (recent illness requiring hospitalization). For the item of familiarity with psychology and related knowledge, we used five scales (hardly, somewhat, average, more, very familiar). The attention to COVID-19 was on a scale of 0–10, which the invitee could adjust by dragging the progress bar according to his or her situation. The item on the impact of COVID-19 had six sub-topics:

- Health behaviors (inconvenience or discomfort caused by wearing masks, washing hands, disinfection, etc.);
- Quarantine policies (policies on measures to deal with the pandemic have limited people’s freedom of travel or reduced their mobility);
- The adverse information, the negative or false information distresses;
- Economic aspect, the price changes or difficulties in employment and income due to the pandemic.
- The medical troubles, many difficulties in accessing medical care, and shrinking medical resources due to the pandemic or prevention and control strategy;
- The mental health, psychological and emotional changes due to the pandemic and containment policies, such as increased self-perceived anxiety, depression, restlessness, insomnia, irritability, etc.

GAD-7 is a quantitative evaluation standard recommended by the 5th edition of the Diagnostic and Statistical Manual of Mental Disorders published by the American Psychiatric Association [26]. The GAD-7 scale is an effective tool to identify possible causes of generalized anxiety disorder [30], which had good reliability and validity in previous studies [31, 32]. According to the GAD-7 score standard, the patients were divided into groups 0–5, 6–9, 10–14, and 15–21, corresponding to no, mild, moderate, and severe anxiety, respectively [26].

PHQ-9 is based on the nine criteria of depression in the Diagnostic and Statistical Manual of Mental Disorders published by the American Psychiatric Association, and it is highly sensitive to changes in depressive symptoms [33, 34]. According to the scoring criteria, PHQ-9 scores were divided into five groups: 0–4, 5–9, 10–14, 15–19, and 20–27, respectively, corresponding to no, mild, moderate, moderate to severe and severe depression respectively.

### Quality control

GAD-7 and PHQ-9 have demonstrated good reliability and validity in previous studies. All investigators have sophisticated experience in epidemiological investigation, the questionnaire is filled out by the subjects themselves, and the ambiguities of the questions investigated are explained by the investigators. To ensure that the study can reflect the specific situation of the participants as much as possible, there were instructions at the cover page, which included the purpose of the research, informed consent form, questionnaire filling conditions, and others, contributing to reduce the bias of the study.

### Ethical approval and consent from participants

This study was conducted following the Helsinki Declaration and China’s relevant clinical trial research norms and regulations [35]. This research project was conducted after obtaining approval from the Ethics Committee of the Tenth People’s Hospital of Tongji University. The clinical information used in this study was obtained with informed consent and conducted under the institutional review boards’ protocols of the participating institutions. Written informed consent was obtained from all participants and/or their legal guardians (participants whose age is less than 16 years). Limited by the online approach, the purpose of the study and the authorized consent related to filling out the questionnaire was informed on the first page of this research questionnaire, and all participants were informed and agreed to participate in this research.

### Statistical analysis

The internal consistency of GAD-7 and PHQ-9 was evaluated by *Cronbach’s* α coefficient, and *Cronbach’s α* ≥ 0.7 indicated that internal consistency was sufficient. Split-half reliability was calculated using the item parity subscale and *Spearman-Brown* formula, and collective validity and discriminant validity were verified using the success rate of the ensemble validity test and discriminant validity test [36, 37]. The questionnaire star was used to export the baseline data and establish a database, and the participants that met the exclusion criteria were excluded into the analysis data. *SAS 9.4* and *SPSS 25.0* software were used for data analysis. The measurement data with normal distribution were described by *x* ± *s*, and the counting and grade data were described by examples and percentages. The *T-test* or *Wilcoxon rank-sum* test was used to compare the two groups, whereas the × *2* test or *CMH* × *2* test was used to compare the two groups of counting data and grade data. After the normality test of the data, the *Spearman* correlation coefficient was used to evaluate the correlation between anxiety and depression scores. Disordered multi-classification *Logistic* regression analysis was used in the multifactor analysis [38–40].

## Result

### Baseline demographics

The questionnaires were distributed to 185 participants. After collecting the filled questionnaires, 6 unqualified questionnaires were excluded, and 179 (96.76%) valid questionnaires entered into the statistical analysis. Table 1 displayed the demographics, which included 77 (43.02%)males and 102 (56.98%) females.

**Table 1:** Baseline demographics and clinical characteristics

	Female (n = 102)	Male(n = 77)
Age, years old		
< 18	1 (1.0)	1 (1.3)
18–25	98 (96.1)	72 (93.5)
26–30	3 (2.9)	4 (5.2)
Recent health status		
Healthy	100 (98.0)	75 (97.4)
Isolated	1 (1.0)	0 (0.0)
Poor	1 (1.0)	2 (2.6)
The attention to COVID-19	7.8 ± 1.9	7.8 ± 2.1
The impact of COVID-19		
(i) Health behaviors	77 (75.5)	47 (61.0)
(ii) Quarantine policies	81 (79.4)	53 (68.8)
(iii) The adverse information	26 (25.5)	17 (22.1)
(iv) Economic aspect	24 (23.5)	18 (23.4)
(v) The medical troubles	23 (22.5)	19 (24.7)
(vi) The mental health	38 (37.3)	31 (40.3)
Familiarity with psychology and related knowledge		
Hardly	4 (4.0)	3 (3.9)
Somewhat	5 (4.9)	9 (11.7)
Average	24 (23.5)	10 (13.0)
More	37 (36.3)	29 (37.7)
Very	32 (31.3)	26 (33.7)
Anxiety classification of GAD-7		
No anxiety	63 (61.8)	58 (75.3)
Mild	26 (25.5)	16 (20.8)
Moderate	7 (6.8)	3 (3.9)
Severe	6 (5.9)	0 (0.0)
Depression classification of PHQ-9		
No depression	52 (51.0)	44 (57.1)
Mild	31 (30.4)	20 (26.0)
Moderate	9 (8.8)	9 (11.7)
Moderate to severe	9 (8.8)	4 (5.2)
Severe	1 (1.0)	0 (0.0)

Normally distributed measures were described by *x* ± *s*, and counts and graded data were described by number of cases and percentages

### Reliability and validity

The Cronbach’s coefficient of the GAD-7 scale was 0.92 and the split-half reliability was 0.90, with high reliability. The Cronbach’s coefficient and the split-half of the PHQ-9 scale were 0.90 and 0.90, respectively, indicating that the PHQ-9 was reliable, as depicted in Table 2.

**Table 2:** Reliability and validity of GAD-7, PHQ-9 scales

Scale	Items	Cronbach’s α	Split-half	Ensemble validity			Discriminant validity		
				*r*_*s*_^a^	Successes/trials	Success rate, %	*r*_*s*_	Successes/trials	Success rate, %
*Validity analysis*									
GAD-7	7	0.923	0.899	0.71–0.85	7/7	100.00	0.77–0.88	7/7	100.00
PHQ-9	9	0.900	0.895	0.58–0.82	9/9	100.00	0.63–0.82	9/9	100.00

^a^*r*_*s*_ range of rank correlation coefficient

The rank correlation coefficient between the item scores and the scale was calculated, and a set validity test was successful when *r*_*s*_ ≥ 0.4. The discriminant validity test was also successful when the rank correlation number between the items and the dimensions was significantly higher than that with other dimensions. The ensemble and discriminant validity of GAD-7 scale and PHQ-9 scale, respectively, were both 100%, demonstrating good validity (Table 2).

### Anxiety and depression

Median GAD-7 and PHQ-9 scores for university students were 2.0 (0.0, 17.0) and 3.0 (0.0, 21.0), respectively. According to the scoring criteria, 121 students (67.6%) had no anxiety symptoms, while 42 (23.5%), 10 (5.6%) and 6 (3.3%) university students had mild, moderate and severe anxiety symptoms, respectively.. 96 of 179 (53.6%) students had no depressive symptoms, while 51 (28.5%), 18 (10.0%), 13 (7.3%) and 1 (0.6%) students had mild, moderate, moderate and severe depressive symptoms, respectively. The correlation coefficient between the GAD-7 and PHQ-9 scores was 0.65 (p < 0.01) by *Spearman* rank correlation analysis, indicating a strong positive correlation between depression and anxiety symptoms of college students with the COVID-19 pandemic.

### Influencing factors analysis

**Fig. 1:**
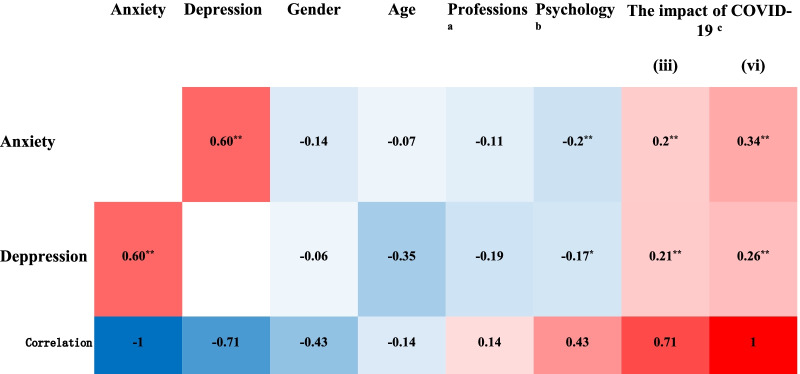
Correlation heat map of anxiety and depressive symptoms with factors. ^a^The medical students of profession; ^b^familiarity with psychology and related knowledge; ^c^the impact of COVID-19 [(iii) the adverse information, and (vi) the mental health]. *P < 0.05; **P < 0.01

To find the influencing factors associated with anxiety and depressive symptoms, we conducted a *Spearman* correlation analysis of anxiety and depression with the entries in the general questionnaire. We found that the impact of COVID-19 ((iii) the adverse information, and (vi) the mental health) were both positively correlated with anxiety and depressive symptoms. Familiarity with psychology and related knowledge were both negatively correlated with anxiety and depressive symptoms (Fig. 1). In addition, depression was negatively correlated with the medical students of profession, probably medical students are more exposed to psychology and related knowledge (*Spearman* correlation: 0.51, *p* < 0.01).

### Subgroup analysis

Furthermore, stepwise regression was used to screen variables, and the results of parallelism test were χ^2^ = 33.78 (p = 0.013) and χ^2^ = 56.05 (p = 0.018). Therefore, two models were established by disordered multi-category Logistic regression: GAD-7 anxiety model and PHQ-9 depression model.

By statistical analysis, with no anxiety symptoms as a reference, during the COVID-19 pandemic period, college students with strong psychological quality and not easily affected by the pandemic were less likely to have mild anxiety symptoms, OR (95% CI) was 0.12 (0.05, 0.33). Secondly, male university students who received less negative or false information and who possessed a strong grasp of psychology and related knowledge were less likely to suffer from moderate anxiety symptoms. The OR (95% CI) values were 0.18 (0.04, 0.81), 0.23 (0.06, 0.89), and 0.07 (0.01, 0.74). College students who were familiar with psychology and related knowledge might also be easy to reduce moderate anxiety, OR (95% CI) were 0.06 (0.01, 1.10) (p = 0.06), 0.15 (0.02, 1.40) (p = 0.09) (Table 3).

**Table 3:** Multinomial-logistic regression analysis of the influencing factors of anxiety

Variables	Mild		Moderate	
	OR (95%CI)	*p*	OR (95%CI)	*p*
Gender				
Female	Ref		Ref	
Male	0.68 (0.30, 1.55)	0.36	0.18 (0.04, 0.81)	0.03*
Grade				
Doctoral student	Ref		Ref	
Postgraduate	3.25 (0.20, 53.15)	0.41	3.51E−9^①^	
Undergraduate	0.68 (0.06, 8.41)	0.76	0.15 (0.01, 2.18)	0.16
The first-grade college student	0.39 (0.03, 6.03)	0.50	9.84E−10 (0.00, +~)	0.99
*The impact of COVID-19*				
The mental health				
Yes	Ref		Ref	
No	0.12 (0.05, 0.33)	0.001**	0.27 (0.07, 1.11)	0.07
The adverse information				
Yes	Ref		Ref	
No	0.79 (0.29, 2.14)	0.65	0.23 (0.06, 0.89)	0.03*
Familiarity with psychology and related knowledge				
Hardly	Ref		Ref	
Somewhat	2.51 (0.18, 35.57)	0.50	0.63 (0.05, 7.71)	0.72
Average	2.56 (0.22, 29.98)	0.46	0.06 (0.01, 1.10)	0.06
More	1.08 (0.97, 12.04)	0.95	0.15 (0.02, 1.40)	0.09
Very	0.35 (0.03, 4.27)	0.40	0.07 (0.01, 0.74)	0.03*

*OR (95%CI)* OR value (95% confidence interval). *Ref*: Reference level. *E*+: multiplied by the positive power of 10. *E−*: multiplied by the negative power of 10; ①: this item has only one person, no confidence interval and p value; +~ : no upper limit; **p < 0.001, *p < 0.05, there is a significant difference between the two groups

Taking no depression symptoms as the control group, college students unaffected by COVID-19 pandemic could easily mediate their emotions and were less likely to suffer from mild, moderate, and moderate to severe depression symptoms. The OR values (95% CI) were 0.23 (0.08, 0.65), 0.22 (0.05, 0.93), 0.10 (0.02, 0.54). Students who were in good health during the pandemic and did not pay special attention to health problems might easily get rid of mild depression and moderate to severe depression {OR [95% CI 1.73 (0.75, 3.99), 3.52 (0.68, 18.16)]}. With being healthy, the students may be able to overcome moderate and severe depressive symptoms with much or little understanding of psychology and its related knowledge [OR (95% CI) 0.11 (0.01, 772), 0.16 (0.01, 1.89), 0.10 (0.01, 1.23)] (Table 4).

**Table 4:** Multinomial-logistic regression analysis of factors to severity of depression symptom

Variables	Mild		Moderate		Moderate and severe	
	OR (95%CI)	*p*	OR (95%CI)	*p*	OR (95%CI)	*p*
The recent health						
Poor	Ref		Ref		Ref	
Isolated	8.53E+11 (0, +~)	0.99	1.52E+5 (0.0, +~)	0.99	0.07 (0.0, +~)	0.99
Healthy	5.9E+5 (0.0, +~)	0.99	1.74E+5 (0.0, +~)	0.99	0.11 (0.0, 1.7)	0.11
*The impact of COVID-19*						
The mental health						
Yes	Ref		Ref		Ref	
No	0.23 (0.08, 0.65)	0.001**	0.22 (0.05, 0.93)	0.04*	0.10 (0.02, 0.54)	0.01*
The adverse information						
Yes	Ref		Ref		Ref	
No	0.57 (0.23, 1.43)	0.23	0.55 (0.15, 2.04)	0.37	0.40 (0.09, 1.81)	0.24
Concerns during the pandemic: health						
Yes	Ref		Ref		Ref	
No	1.73 (0.75, 3.99)	0.20	1.90 (0.54, 6.70)	0.32	3.52 (0.68, 18.16)	0.13
Familiarity with psychology and related knowledge						
Hardly	Ref		Ref		Ref	
Somewhat	0.41 (0.04, 4.96)	0.49	1.37E+7 (0.0, +~)	0.99	5.17E−7 (0.0, +~)	0.98
Average	0.72 (0.08, 6.24)	0.76	3.55E+6 (0.0, +~)	0.99	0.21 (0.02, 2.94)	0.25
More	0.48 (0.06, 3.96)	0.50	2.41E+6 (0.0, +~)	0.99	0.16 (0.01, 1.89)	0.15
Very	0.27 (0.03, 2.30)	0.23	2.08E+6 (0.0, +~)	0.99	0.10 (0.01, 1.23)	0.07

*OR (95% CI)* OR value (95% confidence interval). *Ref* Reference level. *E*+: multiplied by the positive power of 10. *E−*: multiplied by the negative power of 10; +~: no upper limit; **p < 0.001; *p < 0.05, there is a significant difference between the two groups

## Discussion

This study is a cross-sectional survey involving college students at a Shanghai university. The investigation period was from March 9 to March 12, 2020, during the COVID-19 pandemic, and the domestic pandemic situation had greatly improved and was in the retreating period. In contrast, the international pandemic situation was still in a state of rapid growth [4–6]. Previous studies have demonstrated that such public health events were sudden, public attributes, and serious social harmfulness [41]. In contrast, college students lacked experience in dealing with emergencies, were emotionally unstable, could not analyze and make decisions, and were easily affected by the pandemic. Rapid psychological and emotional changes led to some irrational behavioral impulses [42]. If this kind of bad behavior and unhealthy psychology were not solved reasonably, it would have direct or indirect effects on all fields of social security [43, 44]. To prevent the escalation of the pandemic, the opening of colleges and universities was postponed, as were all academic-related matters, and college students were required to go out less, resulting in their inability to study and participate in social activities, which could affect their learning progress and aggravate their anxiety and depression symptoms, and even aggravate their anxiety and depression symptoms by the unlimited suspension of graduation-related matters. Therefore, the mental health problems of college students should be given priority. The purpose of this study was also to understand the psychological status of college students in facing to public health emergency in the form of a questionnaire, to analyze in-depth the causes or protective factors that could lead to a terrible mood, and to provide the corresponding mitigation basis or plan for the problem.

The Cronbach’s coefficient and split-half reliability of the GAD-7 scale and PHQ-9 scale in our study were both greater than 0.85, indicating that their reliability and validity are higher than those of previous studies [33, 34, 45]; This also demonstrated that GAD-7 scale and PHQ-9 scale are suitable for evaluating anxiety and depression symptoms of college students.

Of 179 students in our study, the incidence of anxiety and depression was 32.40% and 46.40%, which are higher than the previous surveys [46, 47]. With the stress state of the COVID-19 pandemic, the incidence of anxiety and depression among college students has increased significantly, highlighting the need for intervention and health education measures to alleviate the anxiety and depression of college students during the sudden outbreak of COVID-19. Compared with other surveys during the COVID-19 pandemic, anxiety and depression were significantly higher than in other surveys [46, 47] but lower than in adolescents [42]; it is possible that the anxiety and depression among college students may be related to the regional distribution, or that the anxiety and depression of college students gradually increased with the prolongation of the COVID-19 pandemic [10, 48].

This study discovered that college students with strong psychological quality who were not easily affected by the COVID-19 pandemic and male students who received less negative or false information had a good grasp of psychology and related knowledge were less likely to exhibit mild or moderate anxiety symptoms. Furthermore, college students who were not affected by the COVID-19 pandemic were less likely to suffer from mild, moderate, and moderate to severe depression symptoms. Therefore, more attention should be paid to the mental health problems of college students who experience mood swings during the COVID-19 pandemic, are vulnerable to false or negative information, and have less contact with psychology and related knowledge. First, further learning revealed that college students who were prone to mood swings and easily affected by false or negative information, and had less contact with psychology and related knowledge were at a greater risk of developing symptoms of anxiety or depression during the COVID-19 pandemic, possibly because they were unable to find a suitable way to relieve their unhealthy feelings. Based on this, government or colleges and universities should improve their routine teaching skills and popularization of knowledge in psychology and related fields. Psychological counseling and psychological lectures are indispensable, especially during the pandemic period, and measures should be taken to address mental health problems, such as providing remote psychological counseling for students. Second, the survey found that females were more prone to anxiety than males because females were more emotional and vulnerable to tension, so psychological counseling in colleges and universities should focus more on females. In short, the influence factors in our study are gender, mental state fluctuation during the pandemic, negative and false information, and exposure to psychology and related courses. The results of our study are consistent with other recent studies [42, 46, 47, 49].

In this study, there may be some limitations. First, this study was a cross-sectional study, and the subjects were college students, the results are not applicable in the context of longitudinal studies or other populations. More studies are needed to understand the dynamic process of anxiety and depression caused by COVID-19, and more studies are needed to determine the specific psychological state and related factors in the people of other areas and occupations. Second, the anxiety and depression symptoms in this study were diagnosed through the universal self-rating scale analysis, which differed from the clinical diagnostic criteria; therefore, the results of this study are only for reference and cannot be used as guidance for clinical diagnosis and treatment. Third, the sample size of our study was 179, and the proportion of moderate and severe anxiety and depression symptoms was relatively small, not all statistically significant influences were necessarily found in the correlation factor analysis; more samples are needed to investigate the characteristics of moderate and severe anxiety and depression.

In summary, government and universities should strengthen monitoring and information management, timely take health education measures to students according to their characteristics of students, and conduct extensive and in-depth health education and health promotion activities so that students can correctly understand the COVID-19, improve their awareness and ability of self-protection, and be guided to study with positive and healthy behavior and mentality [50–52].

## Conclusion

The GAD-7 and PHQ-9 scale are suitable for evaluating anxiety and depression symptoms in college students. With the stimulation of the COVID-19 pandemic, anxiety and depression symptoms of college students generally increased in general, with gender, mental state fluctuations during the pandemic, exposure to negative and false information, and exposure to psychology and related courses being the most influencing factors. Government and universities should focus on the mental health of students to reduce the damage caused by the COVID-19 pandemic. While COVID-19 is still ongoing, specific measures such as mental health education lectures and stress relief courses should be provided to reduce the incidence of anxiety and depression symptoms among college students.

## Supplementary Information

**Additional file 1. **Description of sample size.

## Data Availability

The data that support the findings of this study are available on request from the corresponding authors. The data are not publicly available due to privacy or ethical restrictions.
