# Supplementary material for: Evaluation and analysis of anxiety and depression symptoms for college students during COVID-19 pandemic
Source: BMC Psychol. 2022 Sep 30;10:227. doi: 10.1186/s40359-022-00934-1 (PMC9523640; doi:10.1186/s40359-022-00934-1)
Supplement: Supplementary file 1 — Additional file 1. Description of sample size. [file 40359_2022_934_MOESM1_ESM.docx]

**Supplementary materials**

The sample size was calculated using the normal approximation of a single group rate. Checking relevant data, we derived the incidence of anxiety and depression from approximately 0.2, and we predicted anxiety and depression during the epidemic to be 0.3 based on the effect of previous public events. The sample size was calculated as follows.

$$n=\frac{\left[ \left( U_{1-\frac{\alpha}{2}} \right)\sqrt{p0\left( 1-p0 \right)}+\left( U_{1-\beta} \right)\sqrt{p1\left( 1-p1 \right)} \right]^{2}}{\left（ p0-p1 \right）^{2}}$$

p0: Pre-epidemic prevalence of anxiety and depression about 0.2.
p1: Expected prevalence of anxiety and depression under the epidemic about 0.3 (expected value).
U1-α/2 and U1-β represent the quartiles of the standard normal distribution corresponding to 1-α/2 and 1-β, obtained by looking up the standard normal distribution table (when α=0.05,1-β=0.9, U1-α/2 and U1-β are 1.96 and 1.28, respectively).
n: required sample size.
 By calculation, we would require 188 cases of participants, and in reality, we collected 185 data, which is approximate to our expected sample size. We finally screened the sample to 179 cases due to missing data or unqualified filling quality.
